# More linear than log? Non-symbolic number-line estimation in 3- to 5-year-old children

**DOI:** 10.3389/fpsyg.2022.1003696

**Published:** 2022-10-31

**Authors:** Maciej Haman, Katarzyna Patro

**Affiliations:** ^1^Faculty of Psychology, University of Warsaw, Warsaw, Poland; ^2^Department of Psychology, University of Tuebingen, Tübingen, Germany

**Keywords:** number-line estimation task, log-to-linear shift, non-symbolic numerical representation, spatial-numerical associations, numerical development, preschoolers

## Abstract

The number-line estimation task has become one of the most important methods in numerical cognition research. Originally applied as a direct measure of spatial number representation, it became also informative regarding various other aspects of number processing and associated strategies. However, most of this work and associated conclusions concerns processing numbers in a symbolic format, by school children and older subjects. Symbolic number system is formally taught and trained at school, and its basic mathematical properties (e.g., equidistance, ordinality) can easily be transferred into a spatial format of an oriented number line. This triggers the question on basic characteristics of number line estimation before children get fully familiar with the symbolic number system, i.e., when they mostly rely on approximate system for non-symbolic quantities. In our three studies, we examine therefore how preschool children (3–5-years old) estimate position of *non-symbolic* quantities on a line, and how this estimation is related to the developing symbolic number knowledge and cultural (left-to-right) directionality. The children were tested with the Give-a-number task, then they performed a computerized number-line task. In Experiment 1, lines bounded with sets of 1 and 20 elements going left-to-right or right-to-left were used. Even in the least numerically competent group, the linear model better fit the estimates than the logarithmic or cyclic power models. The line direction was irrelevant. In Experiment 2, a 1–9 left-to-right oriented line was used. Advantage of linear model was found at group level, and variance of estimates correlated with tested numerosities. In Experiment 3, a position-to-number procedure again revealed the advantage of the linear model, although the strategy of selecting an option more similar to the closer end of the line was prevalent. The precision of estimation increased with the mastery of counting principles in all three experiments. These results contradict the hypothesis of the log-to-linear shift in development of basic numerical representation, rather supporting the linear model with scalar variance. However, the important question remains whether the number-line task captures the nature of the basic numerical representation, or rather the strategies of mapping that representation to an external space.

## Introduction

For years, number-line estimation (NLE) procedures have been used as a teaching aid in early mathematical education (Ernest, 1985), however, their use in developmental cognitive science started with Siegler and Opfer (2003) seminal paper. The authors developed two versions of the procedure. The first, called number-to-position (NP) task, requires the estimation of the position of a given number on a physically presented line (e.g., on a sheet of paper or on a computer screen), by putting a mark on it. The line is typically bounded with numbers determining the limits of its numerical range (e.g., 0 and 10 or 0 and 1,000). The second variant of the task is the position-to-number (PN) task, which requires to estimate numerical magnitude of a number whose position is indicated on a (typically) bounded number-line.

Originally, Siegler and Opfer (2003) used NLE task to distinguish between two basic conceptual models of numerical representation: the accumulator model, which assumes a linear scaling of numerical representation with scalar variance (Meck and Church, 1983; Gallistel and Gelman, 2000) and the logarithmically scaled, spatially organized “mental number-line” (or “mental ruler”; MNL model; Dehaene, 1997). Siegler and Opfer (2003) assumed a direct link between the shape of subjects’ mental number representation (logarithmically compressed or linear) and the way they map numbers onto external space, and that the estimation pattern resulting from the number-line task may give a hint to the nature of and developmental changes in the representations of numerical quantities. Their own results supported the multiple representation model, with a developmental trend progressing from a dominant logarithmic mental number-line representation in the youngest participants studied (8-year-olds) to the rising role of the linear model with increasing age and mastery of numerical knowledge. In a series of subsequent studies, Siegler and Booth (2004) and Booth and Siegler (2006) confirmed the log-to-linear shift hypothesis with younger participants. This hypothesis dominated further research into numerical development around the age at which children start school, especially after a series of publications demonstrated that linear estimation on number-lines is a strong predictor of early math success (e.g., Booth and Siegler, 2006, 2008) and that a delayed log-to-linear shift is observed in children with mathematical learning disabilities (e.g., Geary et al., 2008; Sella et al., 2013). Siegler and Booth (2004), as well as a number of authors of subsequent studies, also showed that the mean value of the percent absolute error (PAE) of estimation decreases with development, which also turned out to be a strong predictor of mathematical skills (for meta-analysis, see Schneider et al., 2018).

After the interest in number-line studies exploded, different authors tried to fit the estimation pattern to other mathematical functions than logarithmic and linear. Some authors observed that seemingly logarithmic mapping can be better modeled by two-segment linear function, with one linear component corresponding to the numerical range which subjects master better (e.g., single digits) and the other, with a flatter slope, to the one with which subjects are less familiar (e.g., two-digit numbers; Ebersbach et al., 2008; Moeller et al., 2009; Helmreich et al., 2011; Moeller and Nuerk, 2011). Thus, estimation pattern would give a more direct hint to children’s numerical knowledge rather than to the hypothetical underlying representation. Furthermore, Barth and Paladino (2011) observed that even the linear estimation pattern could be better modelled by a cyclic power function, which is a signature of proportional reasoning. The model predicts that estimation gets most accurate at the line’s endpoint and certain reference points like half of the line, quarter etc., which may give a rough picture of a linear pattern. Barth and Paladino (2011) argued that such a function likely results from utilization of proportion judgment by older children and adults rather than direct mapping the estimated magnitude. Children who yet lack basic arithmetic and mensuration skills to perform such a reasoning, may “naively” place numbers on a line disregarding value of its right endpoint, resulting in logarithmic-like compression (Barth et al., 2011; Cohen and Blanc-Goldhammer, 2011; Opfer et al., 2011; Slusser et al., 2013; Cohen and Sarnecka, 2014; Link et al., 2014; Dackermann et al., 2015; Cohen and Quinlan, 2018). Other researchers attributed estimation patterns to response biases, like the anchoring effect or making reference to previous trials (Anobile et al., 2012; Cicchini et al., 2014). In Karolis et al. (2011) study, depending on the version of the task, adult participants either underestimated or overestimated the spatial quantities corresponding to large numbers. Based on all this evidence, it may be suggested that the estimation error appears only at the stage of mapping to the external space, indicating that the relation between mapping from the number’s inner spatial representation onto external space may be indirect (but see Kim and Opfer, 2017, 2018, 2020 for the defense of the concept of a logarithmically scaled number line as the basic numerical representation and log-to-linear shift in the NLE, and Cohen and Ray, 2020, for discussion).

Despite this lack of consensus, we believe that the NLE task may be informative about certain basic properties of subject’s numerical representations. First, the fact that even youngest children regularly (though yet inaccurately) arrange numbers along a line may suggest that they posses some idea about numerical ordinality (i.e., that numbers can be arranged in a sequence) and intervals (i.e., that numbers are always at a certain distance from each other), followed by a successor function (any subsequent number is distant exactly 1 unit from the previous one). Second, the NLE requires understanding that numbers and space are related. In particular, intervals between numerical magnitudes need to be converted to spatial distances, and numerical sequences need to be arranged as increasing consistently in one direction. Even though in most research only canonical left-to-right number line is used, some authors which manipulated directionality of the line found that line orientation may indeed be relevant for estimation accuracy (Ebersbach, 2015; Di Lonardo et al., 2020).

Nevertheless, one may doubt whether the NLE task, especially in the typical, symbolic version, actually reveals the scaling of the elementary representation of numerical magnitudes. The symbolic number line itself is a mathematical concept which children use and are exposed to from the beginning of their math education. More indicative could be the results of the estimation of the number line in children before formal mathematics education, using non-symbolic representations of the number.

Even uneducated subjects, such as some animals and young children (including infants) posses ability to process numerical magnitudes in non-symbolic format, *via* the so called approximate number system (ANS; Feigenson et al., 2004; Odic and Starr, 2018). It has been widely debated how both systems–non-symbolic and symbolic ones–are related to each other. Many authors proposed that meanings of symbolically represented numbers are actually grounded in the primitive approximate number system (Dehaene, 1997; Nieder, 2020), but there are also studies which suggest that the relations between both systems may not be so close (Goffin and Ansari, 2019). Crucially, different authors also report conflicting results regarding correlation between symbolic and non-symbolic NLE (e.g., Sasanguie et al., 2013; Yuan et al., 2020). Irrespective of the debate, there are good reasons to claim that the systems at least share certain properties which may be fundamental for our numerical thinking, and particularly for the ability to convert number magnitudes into number-line representation. First, even approximate magnitudes can be arranged in a certain increasing order, from the smallest to the largest one, and sensitivity to certain numerical orders of non-symbolic numerosities has been observed already in infants (de Hevia et al., 2014; McCrink and De Hevia, 2018). Second, ANS enables at least approximate operations of addition and subtraction (Gilmore, 2015; Haman and Lipowska, 2021), as well as comparison of differences between two sets (Patro and Haman, 2012; Odic, 2018), which gives a general idea that numerical values are somehow distant from each other and these distances vary in a regular manner. Finally, both symbolic and non-symbolic numbers can be processed spatially (for review and discussion, see Patro et al., 2014; Toomarian and Hubbard, 2018). In particular, numerical values can be associated with spatial extent like line length or size, or spatial distance (larger values correspond to larger spatial extent; Lonnemann et al., 2008; de Hevia and Spelke, 2009, 2010). They can also be associated with certain directions in space (e.g., small numbers/numerosities with the left side, large numbers/numerosities with the right side; Opfer and Furlong, 2011; Adachi, 2014; Rugani et al., 2015; de Hevia et al., 2017; McCrink et al., 2017; Rugani and de Hevia, 2017; Di Giorgio et al., 2019).

Nevertheless, one should keep in mind that the ANS lacks accuracy of the symbolic system. The properties which ensure equidistant relations between successive numbers are acquired only with the symbolic number system. The first step into understanding such relations is learning counting principles, which occur during preschool age (between 3 and 5 years; Le Corre and Carey, 2007; Sarnecka and Carey, 2008). The process of constructing an exact symbolic numerical representation is gradual. It begins with mapping successive small numbers (1–4) to number words. Children at this stage are referred to as “subset-knowers.” Upon reaching the limit of the accurate small number perception system (typically around the age of 4 years), children discover the cardinality and successor principles, thus gaining the possibility of associating any numeral in a sequence with the corresponding quantity. These children are referred to as “CP-knowers” (where “CP” stands for “cardinality principle” or “counting principles”). The level of knowledge of the counting principles is typically determined with the “Give-a-number” procedure (Wynn, 1992; Le Corre and Carey, 2007; Sarnecka and Lee, 2009; Lee and Sarnecka, 2011). Interestingly, some contemporary works suggest that the acquisition of a symbolic number system, in which a constant unit and order are represented explicitly, may lead to changes in the basic system of numerical representation, for example by increasing its precision or linearity of scaling (Mussolin et al., 2014; Shusterman et al., 2016; Lyons et al., 2018; Goffin and Ansari, 2019). Therefore, it is important to see whether and how the NLE can be performed with non-symbolic quantities in this particular period of life, and how it is related to gradual acquisition of the exact verbal system and counting principles.

### Studies on non-symbolic number-line estimation

Surprisingly, only a few out of hundreds of NLE studies used non-symbolic stimuli (e.g., dot arrays), although the non-symbolic (in addition to the symbolic) task was reported just in the original paper by Siegler and Opfer (2003). Studies conducted with preschool children are even more scarce and their results are mixed. Some authors report a linear fit within the 0–10 range and log-to-linear shift within the 0–100 range (Sasanguie et al., 2012a,b, 2016), some logarithmic mapping function (for 0–30 range, Kim and Opfer, 2018; for 0–100 range, Praet and Desoete, 2014; Lee et al., 2022 for even larger ranges), and others two-segment linear model fit (Ebersbach et al., 2008). Interpretation of these studies is additionally hampered by the fact that some of them used a dual format of numerical stimuli presented as visual sets accompanied by number-words or digits.

From our perspective, most important are studies with subjects who have more limited access to education than kindergarten children. Some evidence comes from studies on illiterate indigenous tribes. Dehaene et al. (2008) found that non-symbolic number line estimation of the indigenous Amazonian Mundurucu were skewed toward the logarithmic model even in the 1–10 range, resambling the performance of American preschoolers. Mundurucu have only limited access to education and their native language has no words for exact cardinalities above three. In contrast to this finding, Núñez et al. (2012) observed that adults from another tribe group—the Yupno—consistently mapped them onto the left or right endpoint of the line (see also Cooperrider et al., 2017).

Sella et al. (2015, Experiment 1) studied preschoolers (5–6 years old) and first and third graders, using three versions of quantitative materials with the NP task on a 0–100 scale: symbolic, non-symbolic and continuous quantity. While in the case of continuous quantity, linear mapping was dominant in all age groups, a typical log-to-linear shift was observed for numerosities and symbolic numbers. However, the authors also noticed that some children used linear strategy in the case of non-symbolic numbers, based on continuous visual cues, as in the case of continuos quantities. Experiment 2 showed that log-to-linear shift is specific to numbers, both symbolically represented, and sets, as long as they are not associated with explicit continuous cues.

Yuan et al. (2020) showed that both spread-out dot arrays and symbolic numbers (but not clustered dot arrays) resulted in a logarithmic mapping in children aged 4–6 years, and their estimation errors were related to the children’s counting skills. Importantly, number-to-position estimation and PAE for symbolic numbers were strongly correlated with the estimation of spread-out dot arrays (which is at odds with the results of Sasanguie et al., 2012a,b) and only weakly correlated with clustered dots. According to the authors, the first two tasks test the processing of numerical quantities, while the third is based on proportional reasoning.

In Van’t Noordende et al., 2018 study 3.5–5 years olds used either a local (maintaining proper order relations between successive numbers) or a global (scaled number line) strategy. Interestingly, regardless of the strategy, the degree of its advancement corresponded to traditional measures such as log-to-linear shift or PAE decrease.

Finally, Kolkman et al. (2013a) used the 0–100 NLE task in longitudinal design in both symbolic and non-symbolic versions in 4–6 years old children. They found linearity of estimation clearly higher in the non-symbolic task than in the symbolic one, which additionally justifies the use of non-symbolic materials in the age group we are interested in.

To sum up, few studies using non-symbolic stimuli in NLE tasks seem to support the general presumption about the affinity of numerical representation to spatial ones, albeit with mixed results regarding the pattern of the number-to-space mapping and its developmental trajectory. Unfortunately, most of the studies described above raise some doubts. The ranges 0–1,000 or even 0–100 far exceed the numerical knowledge of young children. Moreover, the estimation of the numerosity of large, dense sets in this numerical range may be based more on spatial (density and area) than numerical cues, especially in younger subjects (Gilmore et al., 2013; Sella et al., 2015; Cicchini et al., 2016; Zorzi and Testolin, 2018).

Another doubt concerns zero as the line lower delimiter, which was used in the original study by Siegler and Opfer (2003) and copied in most of the studies, also those using the non-symbolic materials. It is far unclear whether zero is directly represented in the ANS (Wynn and Chiang, 1998; Nieder, 2016; Krajcsi et al., 2021). Until school age children may not be able to decide which set, one-element or an empty set, is smaller (Merritt and Brannon, 2013). If zero is not part of the basic system of number representation in children, they can shift the estimates toward the better-defined end, producing illusory log scaling. It is worth noting that the study that most strongly questioned the logarithmic estimation of the non-symbolic number line (Ebersbach et al., 2008) used a line starting with 1.

### Aims of the current study

To sum up, little is known about how NLE task is performed with non-symbolic numerosities (visual sets), in the key period of development when symbolic number concepts start to be gradually acquired (i.e., early preschool age). Therefore, we adapted the NLE task to use it with a non-symbolic numerical format in 3- to 5-years-old children. We were interested, on one hand, what estimation pattern (logarithmic, linear, decomposed linear, cyclic power, ets.) is most characteristic for such approximate magnitudes in this age group. Next, we were interested if and how this pattern would change along age and development of exact numerical representation, which is acquired in this age mostly over verbal counting. Since verbal numerical representations implements such properties like ordinality, successor function, cardinality, and numerical equidistance, it may potentially enhance the accuracy of numerosity-to-line mapping. Finally, we also wanted to check if in the NLE task the preschoolers follow the directional left-to-right spatial-numerical mapping rule, shown in other numerical tasks.

We conducted three experiments. In the first experiment, we used the task in a typical “number-to-position” version, with the ends of the line marked with 1- and 20-element sets. The study was divided into two separate sessions with different number-line orientations (left-to-right and right-to-left). The results were analyzed using various indicators, both those used in earlier research on estimation of number-lines (particular model fit and PAE) as well as their variations developed for the purposes of the current study. To reduce task requirements and other limitations which could not be eliminated in Experiment 1, we conducted Experiment 2, using the left-to-right oriented line scaled 1–10. Finally, in Experiment 3, we used the position-to-number (PN) version of the NLE task, again in the 1–20 range. The PN procedure reverses the mapping direction, requiring the numerical magnitude to be tied to the perceived spatial extent, which may block the use of some strategies that do not directly refer to the numerical magnitude representation.

Importantly, we designed our research to avoid the methodological problems mentioned in the previous sections. We did not use zero as delimiters of the line, nor very large and dense sets of elements. In our studies we independently took into account two developmental measures: mastery of the counting principles and age, which allowed at least partially to separate general developmental effects from the effects related to the acquisition of numerical concepts. Based on such design, we formulated the following predictions:

If preschool children are able to map non-symbolic quantities onto a line while preserving a fixed order of numerals and certain distances between them, we would expect that their estimation pattern can be modeled by any function which was used before to model NLE (at least logarithmic), and which assumes ordinal and interval relations. However, if the basic mental representation of the numerical magnitudes takes a form of the logarithmically scaled mental number line, it is expected that at least the youngest or least numerically competent children will show a logarithmic pattern for estimating positions on the number line. A better fit of a linear or cyclic power function may not be diagnostic for the permanent representation scaling, and may indicate that the mapping of numerical magnitudes onto space, although crucial for the processing of numerical information, is not constant, but constructed in the context of a specific task.

If such mapping involves also a directional component, we would expect that precision of estimation is higher for left-to-right oriented line (consistent with cultural background as well as default orientation documented in infants and animals) than right-to-left oriented line.

If such mapping is enhanced by acquisition of verbal numerical system (i.e., understanding principles of counting), we would expect that precision of estimation is higher among children qualified as CP-knowers than those qualified as subset-knowers independently of other developmental factors correlated with age.

## Experiment 1: 1–20 number-lines oriented from left-to-right and right-to-left

### Methods

#### Participants

Sixty-three children (31 female, age range 3.29–5.26, *M* = 4.28, *SD* = 4.50) recruited from four preschools in Warsaw participated in the experiment. The children represented various social backgrounds, but were mostly middle-class. One additional child was rejected due to being unable to follow the instructions for the use of the touchscreen. Written informed consent was collected from the parents of all children who participated in the study. Additionally, all children were verbally asked if they agreed to participate and were allowed to resign at any time during the experiment. Also, if the experimenters observed that the child felt uncomfortable, they were obliged to terminate the study or to propose some rest. The procedures were accepted by the research ethics committee of the Faculty of Psychology at the University of Warsaw and conformed to the requirements of the Declaration of Helsinki.

#### Procedure

The procedure consisted of two sessions. In the first session, the child performed the Give-a-number task and one of the NLE tasks [every second child performed the left-to right (LR) version and the others performed the right-to-left (RL) version]. In the second session (3 to 14 days later), the children performed whichever version of the NLE task they had previously not done. Twenty children were absent from preschool during the time window for the second session or refused to participate again in the study, so their responses were not included in the comparison between the tasks; however, they were used in general analyses (8 children did not take a part in the LR task and 12 in the RL task).

#### Give-a-number

In the Give-a-number task, children were asked to “feed” a puppet with *N* small plastic carrots. One plate with 12 carrots was located behind the child while the other plate was placed empty in front of the puppet opposite the child. After the child put the carrots on the puppet’s plate, the experimenter asked them to confirm that there were indeed *N* carrots. If *N* > 4, the child was encouraged to count to check the number of carrots. If the child succeeded in providing *N*, they were asked to give the puppet *N* + 2 carrots. Otherwise, the child was tested for *N*–1. The procedure started at *N* = 2 and ended at either *N* = 9 or *N* = 10 or when the child failed at *N* + 1 twice, having twice succeeded at *N*. The largest *N* for which the child gave the correct answer twice was the child’s score. This procedure allows discriminating between subset-knowers and CP-knowers (Le Corre and Carey, 2007), with some level of uncertainty in the case of “four-knowers” and “five-knowers” (*cf*. Lee and Sarnecka, 2011). In our study, we assumed N = 5 as a threshold.

#### Number-line estimation task

13-inch (1,366 × 780 pixels) touchscreen laptop with Windows 7 was used. The screen was turned toward the child so that they did not have access to the keyboard. The experiment was scripted with EPrime 2.0 software.

The materials consisted of circles (yellow, with a diameter of 163 pixels) containing sets of small rectangles. Four packages of sets, differing in spatial parameters and the color of elements, were generated using an algorithm designed by Gebuis and Reynvoet (2011).

The procedure started with three training series. At the beginning, a screen was displayed containing a black line, 1,160 pixels long and 8 pixels thick, extending horizontally at 25% of the screen’s height. At its ends, two circles (diameter 163 pixels) containing 1 and 40 elements were displayed. The child was told a story about a boy/girl (Ann or Tom, depending on the participant’s gender) who places plates with cookies (circles containing sets of rectangles) on the table (line) so that on one side there are the fewest cookies and on the other side the most cookies. Then, centrally at the top of the screen, a new set (“cookie plate”) of 10, 20, or 30 elements appeared and the child was asked to show where the protagonist should put this plate. The experimenter confirmed the choice or, if the child indicated the wrong line segment (an error of over 25%), explained, with reference to the sets at the ends of the line: “There are more than here, but less than here, so we’ll move the plate a little closer to there.” The circle was moved to the correct position (according to the linear scale) and another “plate” appeared above the line, with the previous plate persisting on the screen. The whole procedure was repeated two more times, except differing in that when an incorrect line segment was indicated by the participant, the experimenter referred to the nearest two sets, regardless of whether they were the end-points.

The next training series was performed similarly, except that new spatial arrangements of the sets were used and the experimenter corrected the child’s choice only for the first attempt. In subsequent attempts, if the child indicated an incorrect line segment, the experimenter referred to the neighboring set, asking “Are there more here or here? Where do you need to move this plate?” The third training series was arranged in the same way as the test series, except that, as in the previous series, the numbers 10, 20, and 30 were tested. The experimenter informed the child that they would now arrange the plates alone, without help, asking “When a new plate appears at the top, show where the girl/boy should put it.” When the child touched the screen, the set moved to the line at the point where the X coordinate was identical to the indicated one and remained there for 1 s before another trial was displayed. The position of the plates was not corrected and only one plate could be placed on the line at a time. The X and Y coordinates of the indicated location were saved in the output file.

**Figure 1:**
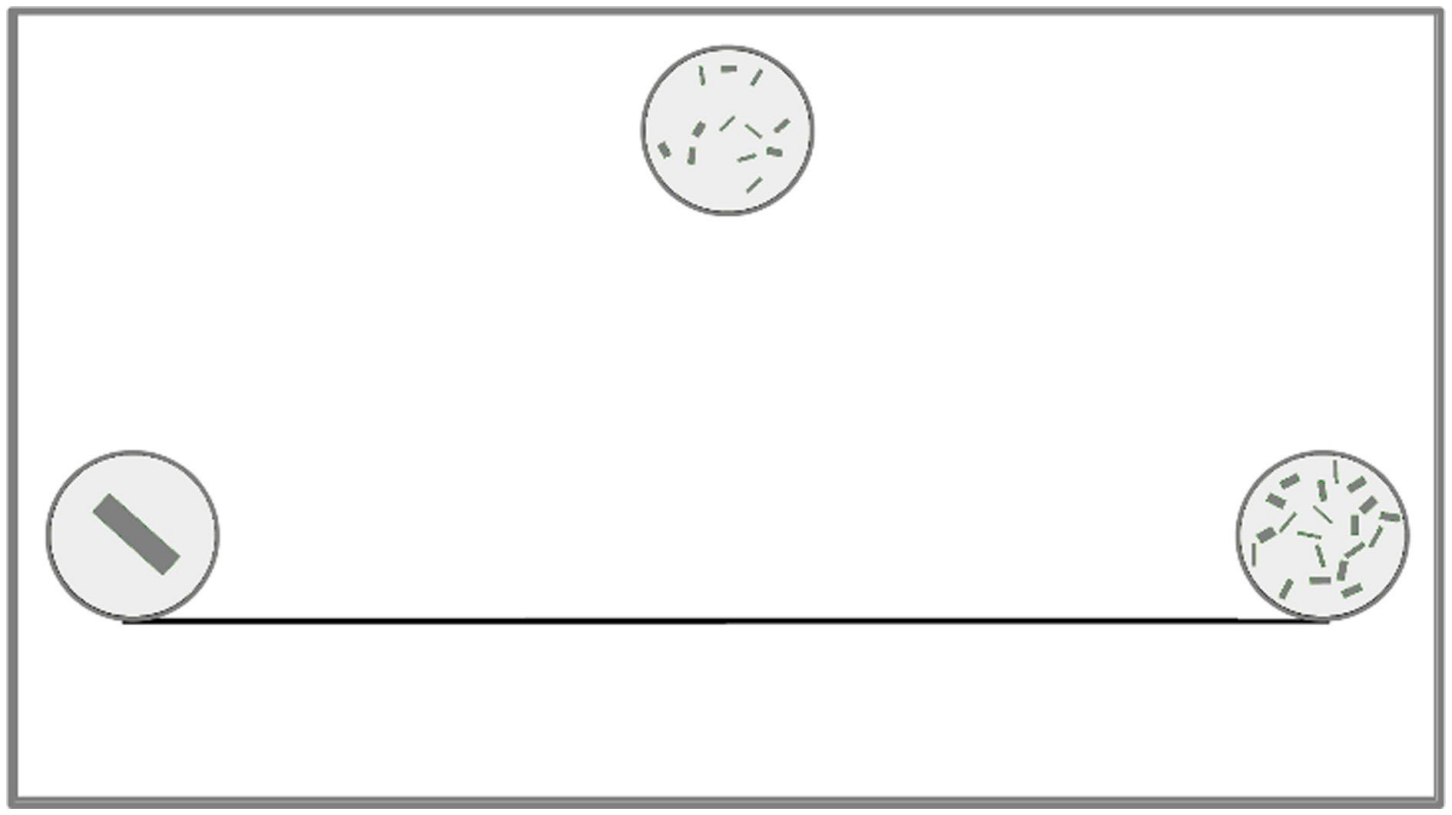
Experiment 1: test trials screen layout: 1–20, left-to-right oriented line.

After training, the ends of the axes were marked with new sets of 1 and 20 elements. The experimenter told the child that they were new plates, asked them where there were fewer and where there were more cookies, and repeated the instruction about placing a new plate that appears at the top. After confirming that the child was ready, the child then performed 18 test trials with sets of 2 to 19 items in random order (see Figure 1 for a sample test screen). After this series was completed, the end-of-line sets were exchanged for new items differing in terms of color and spatial properties, and the entire procedure (except for the training) was repeated.

Different packages of sets (with different distribution and colors of elements) were used in each series, but for each child, the same packages were used to test the estimates in both line directions, to make the comparison reliable. After the second series, the experimenter rewarded the child verbally and allowed them to choose a sticker.

### Results

#### Give-a-number task

Thirty-five children scored below 5 and were therefore classified as subset-knowers (Give-a-number range: 1–4, *M* = 2.5, *SD* = 0.79, age range: 3.29–4.93, *M* = 4.07, *SD* = 0.47). Twenty-three children in this group passed both the LR and RL tasks, six passed only the LR task, and six passed only the RL task. The remaining 29 children scored at least 5 (range: 5–10, *M* = 7.5, *SD* = 1.67, age range: 3.78–5.26, *M* = 4.53, *SD* = 0.43) and were classified as CP-knowers. Twenty children passed both the LR and RL tasks, six passed only the LR task, and two passed only the RL task.

#### Number-line estimation task

##### Data preparation

**A**ll trials in which the child indicated a position in the top 30% of the screen were excluded from the analysis (sometimes children tried to drag the “plate” instead of pointing to the line). That applied on average to 11.21% of subset-knowers choices and 8.45% of CP-knowers choices. However, for all children the number and distribution over line of correctly indicated items was sufficient to calculate the model fit. Next, the best-fit linear and logarithmic regression models were computed on mean of estimated positions of tested numerosities from two runs individually for each participant for the left-to-right and right-to-left oriented lines. In most studies to date, the coefficient of determination (*R*^2^) has been used as a measure of the estimation pattern. The *R*^2^ coefficient, however, is insensitive to the direction of the slope of the regression line, so a model negatively correlated with the data would make a positive contribution to the explained variance. For this reason, results with a negative slope, or even all *R*^2^ non-significantly above 0, have usually been removed from analyses as outliers (Slusser et al., 2013; Sella et al., 2015). However, it should be noted that *R*^2^ is negatively correlated with the estimation variance. With a large variance the regression slope can randomly drop below zero. This can lead to large data loss, and overestimation of a given model fit in remaining part of the sample. On the other hand, a negative regression slope may indicate that the child mentally flips the number line, which may be the case especially with the right-to-left axis. In order to obtain a compromise between these two possibilities, we decided to exclude from the analysis only those children whose regression slope was significantly below zero, while in the case of the remaining children, the sign of the regression slope (“+” or “−”) was added to *R*^2^.

##### Directionality of the line

2 × 2 ANOVA with line orientation (LR vs. RL) and model type (linear or logarithmic) as within-subject factors, and CP-knowledge level (subset-knower vs. CP-knower) as a between-subject factor on the dependent measure described above was run to determine the role of line orientation. Only children who participated in both sessions (LR and RL) were included (N = 41; two additional children were excluded because of significantly negative slope). Only the effect of model type was significant [*F*(1,39) = 12.10, *p* < 0.005, η^2^_p_ = 0.237]. The linear model was generally better fitted than the logarithmic one (*M*_lin_ = 0.301, *SE* = 0.047, *M*_log_ = 0.268, *SE* = 0.043). Neither line orientation, CP-knowledge level nor interaction approached significance (largest *F*[1,39] < 1.92, all *p*s > 0.17).

To validate the null effect of line direction, we repeated this ANOVA using a Bayesian approach with JASP software v. 0.10.02 (JASP Team, 2019). For the line orientation factor, as well as for all interactions involving this factor, the analysis provided at least moderate evidence for the null hypothesis (BF_01_ = 5.899 for a model containing only the orientation factor and all BF_01_ ≥ 4.614 for any interaction model including a line orientation factor). Line orientation was then found not to influence the fit of either model. Assuming that we merged both sessions (LR and RL line orientations), which allowed us to include all cases in subsequent analyses and to base participants’ mean estimates of each numerosity on more trials.

##### Linear and logarithmic model fit

Entire sample (N = 61 after excluding 2 children with significantly negative slope) was included into 2 × 2 ANOVA with individual fit to the model (*R*^2^ with assigned slope sign) as a dependent measure, model type (linear vs. logarithmic) as a within-subject factor, and CP-knowledge level as a between-subject factor revealed prevalence of the linear model over the logarithmic model [*M*_lin_ = 0.303, *SE* = 0.043, *M*_log_ = 0.237, *SE* = 0.039, *F*(1,59) = 16.61, *p* < 0.001, η^2^_p_ = 0.220]. There was also tendency toward better fit of both models in CP-knowers [*M*_subset-knowers_ = 0.207, *SE* = 0.056, *M*_CP-knowers_ = 0.363, *SE* = 0.059, *F*(1,59) = 3.68, *p* = 0.06, η^2^_p_ = 0.059], but no interaction with the modelling function [*F*(1, 59) = 1, *p > 0*.32]. To make our results comparable to other studies, in Supplementary material we also show analyses with only those children who showed a positive regression slope. These analyses replicated the effect reported above.

**Figure 2:**
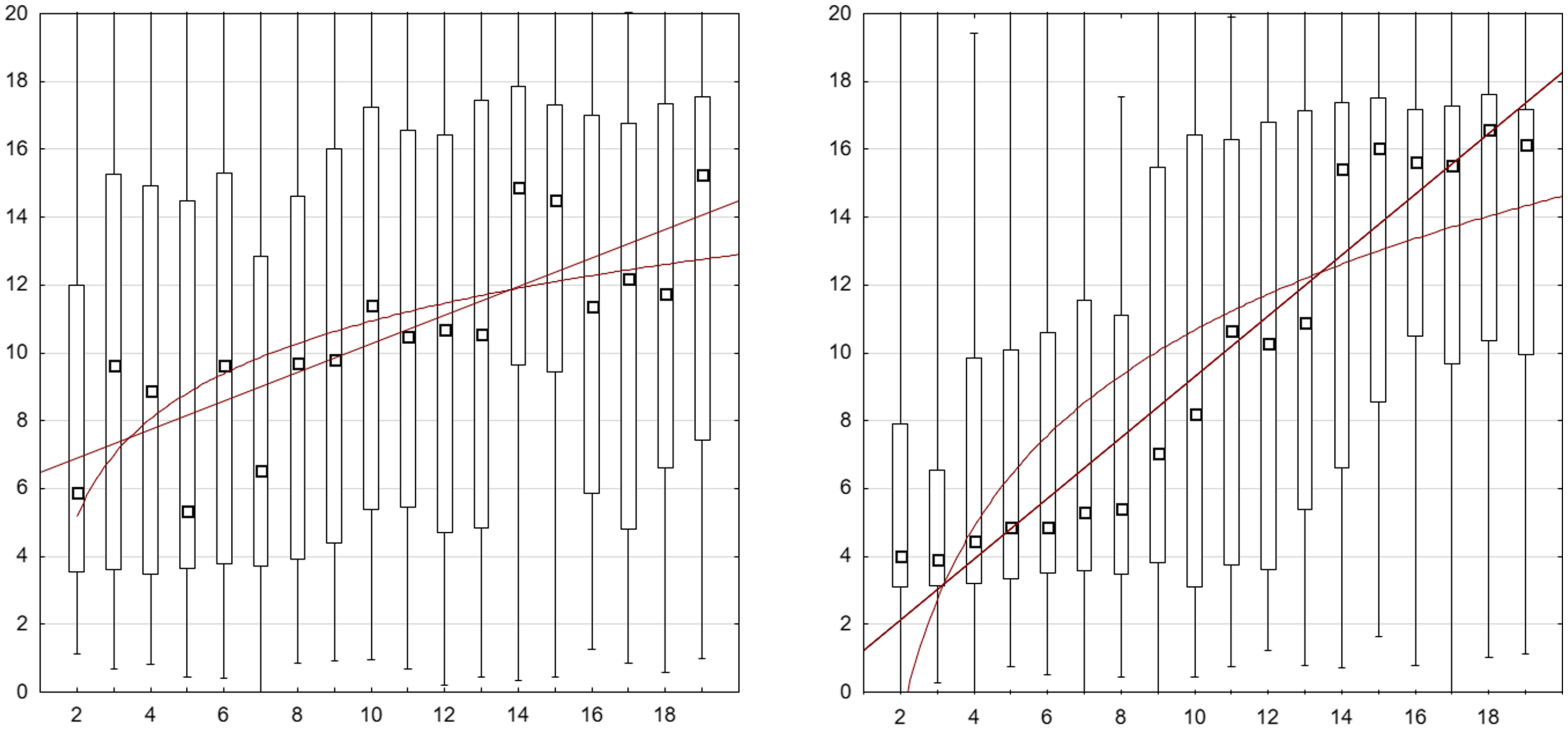
Experiment 1: Distribution of estimated positions on a line based on single data from two runs in both directions of the line. The black square represents the median, the box contains 50% of the middle choices, the bars represents the full range of choices. Left graph: subset-knowers, right graph: CP-knowers. Best fit linear and logarithmic functions plotted on both graphs. For distribution and model fit of group medians in division onto CP-knowledge level and line orientation see Supplemental material.

In line with previous studies we also checked the fit of both models at the level of group medians. Assuming that the differences in the spatial parameters of the sets and in the orientation of the lines may lead to some shifts of the estimated position, we calculated medians for each numerosity separately in each run, which allowed to include 72 measurement points (4 for each numerosity) into the model. The *R*^2^ values for both models were relatively large (entire sample: *R*^2^_lin_ = 0.855, *R*^2^_log_ = 0.767, CP-knowers: *R*^2^_lin_ = 0.815, *R*^2^_log_ = 0.699, subset-knowers: *R*^2^_lin_ = 0.497, *R*^2^_log_ = 0.451; see Figure 2). Based on the method of Siegler and Booth (2004), we compared the quality of fit of both models using paired *t*-tests on the absolute value of the model rests (difference between the median of estimates for a given position and predicted by model). Again, the linear model turned out to be a better fit, at least for entire sample [*t*(71) = −4.29, *p* < 0.001] and CP-knowers [([71) = −4.84, *p* < 0.001], but not for subset-knowers: *t*(71) = −1.08, *p* = 0.3].

We also conducted more detailed analyses taking into account the level of CP knowledge and line orientation. As the results of these analyses only confirm the conclusions of the individual fit analyses presented above, we have placed them in the figures included in Supplementary material.

While comparing the fit of logarithmic and linear models is of the greatest importance to our research questions, we also checked to what extent our participants’ performance could be modeled by a cyclic power function. For this purpose, we adapted the formulas of the Barth and Paladino (2011) models with one and two cycles to the 1–20 scale. Of these two models, the model with one cycle with free parameter (not assumed line end points) turned out to be best fitted (*R^2^*_licpf_ = 0.833). Nevertheless its fit was below the linear model [*t*(71) = 3.50, *p* < 0.001], although above the logarithmic one [*t*(71) = −3.01, *p* < 0.005].

Taken together, all results up to now attest to the fact that even subset-knowers reveal a certain level of regularity in their estimations of the position of sets’ numerosities on a number-line and that the linear model generally explains this regularity better than the logarithmic or cyclic power models.

##### Unitary vs. segmented number-line

Next, we checked whether some multi-segment models explained the obtained data better than a homogeneous linear or logarithmic model. First, we took into account that the estimation of the units near the ends of the lines may differ from the middle part. In the case of small numerosities, this may be due to the separate mechanism of perception of small numbers. Numbers in the range 1–4 can be accurately identified by a subitizing mechanism independent of the ANS. Moreover, the relatively large diameter of the “plates” could cause the children to place the numbers at the ends of the tested range in such a way that they do not overlap with the “plates” marking the ends of the line, which leads to flattening the estimates at both ends. A visual inspection of Figure 2 may suggest such a pattern, so we decided to check if the middle line segment (5–16), with excluded extreme numerosities, would better fit any of the tested models than the entire range. Contrary to this hypothesis, the fit coefficients based on median estimates were in each case significantly lower than for the entire range. Even though the estimation of near-line end numerosities was a bit disturbed, the children seem to use a constant estimation function rather than separate scales for the middle and end line segments. Nevertheless, the linear model still significantly prevails over the logarithmic model for at least the entire sample and CP-knowers (see Table 1 for detailed data).

**Table 1:** Linear and log model fit (*p* for differences determined by paired *t*-test) in the whole range (2–19), the single-middle-segment range 5–16 and separate ranges 2–10 and 11–19, and comparison of the linear model in the range 5–16 with the log model in the range 11–16 for the entire sample and individual CP-knowledge levels.

	Model by range				Paired *t*-tests Lin model 5–16 vs. Log model, 11–19 in the 11–16 range
CP-knowledge level	2–19	5–16	2–10	11–19
	Lin*R*^2^	Log *R*^2^	Lin*R*^2^	Log*R*^2^	Lin*R*^2^	Log*R*^2^	Lin*R*^2^	Log*R*^2^
	*p* (difference)		*p* (difference)		*p* (difference)		*p* (difference)	
All	**0.855**	0.757	**0.819**	0.794	**0.640**	0.529	0.433	**0.453**	*t*(23) = −2.63, *p* <. 015
	*p* < 0.001		*p < 0*.021		*p < 0*.03		*p < 0*.003		
CP-knowers	**0.815**	0.699	**0.703**	0.662	**0.433**	0.357	0.410	0.423	*t*(23) = −0.30, *p* = 0.77
	*p* < 0.001		*p* < 0.04		*p* < 0.041		*p* = 0.14		
Subset-knowers	0.497	0.451	0.451	0.448	0.156	0.143	0.190	0.199	*t*(23) = −1.15, *p* = 0.27
	*p = 0*.3		*p = 0*.82		*p = 0*.72		*p = 0*.3		

Bold: significantly better fitted model; *p* < 0.05.

Some previous studies in preschool children also suggested that the logarithmic-to-linear shift may occur at around the number 10 (Sasanguie et al., 2012b). Most CP-knowers can count up to at least 10, which may increase the accuracy and linearity of scaling the line in this range. We tested this by fitting the linear and logarithmic model separately in the ranges 2–10 and 11–19. For both ranges, the fit indices turned out to be clearly lower than for both the entire line and the middle segment (5–16), but indeed in the case of range 11–19 the logarithmic model turned out to be slightly better fit than the linear one (Table 1). This would suggest a “log-to-linear shift” depending on the scale of the lines. However, the fit of the models for separate segments was generally low. Note also, that flattening of the estimates at the ends of the lines mentioned in the previous paragraph can lead to an illusory exponential fit for smaller numbers (2–10) and an illusory logarithmic fit for large numbers (11–19), which is at odds with a generalized log model. In order to check this possibility, we compared the absolute size of the rests of estimation medians from those predicted by the linear model for the 5–16 segment and both models for the 2–10 and 11–19 segments (including to this comparison only the numerosities present in both compared ranges, thus excluding numbers close to the line ends). The uniform linear model for the 5–16 range was significantly better at predicting children’s estimates, also when compared to the log model in the 11–19 range (although the difference was not significant for any CP-knowledge level separately, but only for the entire group; see Table 1).

##### Scalar variance of estimates

Observing that the linear model fits the data better than the logarithmic model, we considered the possibility that our results may support a number representation model which assumes linear scaling with scalar variance. If this hypothesis is true, a positive correlation is expected between the average variance (or standard deviation) of an estimated position of a given numerosity and a number to be estimated. This hypothesis was not confirmed. The variance, calculated separately for the LR and RL conditions and then averaged, only weakly correlated with the estimated numerosity (*r* = 0.1, *z* = 0.095, *p* = 0.71). Correlations for the LR (*r* = −0.08, *z* = −0.081, *p* = 0.75) and RL (*r* = 0.2, *z* = 0.203, *p* = 0.43) components were also insignificant. And, as Figure 2 shows, the differences of variance for the individual numerical quantities were small and randomly distributed along the line. However, it should be noted that the indicator used may not be sensitive enough, as several factors may influence variance of estimates in this task, especially flattening of estimates near the midpoint and endpoints).

##### PAE analyses

Another commonly-used measure of performance on the NLE task is PAE of estimation, computed according to the formula proposed by Siegler and Booth (2004): (**|**Estimated position-Tested number**|** / Line scale ^*^ 100%). Again, 2 × 2 ANOVA on the subset of those children who performed both number-line tasks, with line orientation (LR vs. RL) as a within-subject factor and CP-level as a between-subject factor, revealed no significant effect of line orientation [*F*(1,40) = 0.010, *p* > 0.75] nor its interaction with CP-level [*F*(1,40) = 0.006, *p* > 0.8]. Thus, we merged both line directions, which allowed us to include more cases into analyses. Independent-sample *t-*test indicated this time significantly lower PAE [*t*(59) = −2.31, *p* < 0.025] in CP-knowers (*M* = 26.9, *SD =* 7.27) than subset-knowers (*M* = 32.0, *SD =* 9.38).

##### Developmental effects

When interpreting the effects of CP knowledge level we should consider that this factor is strongly dependent on age, so it is not clear whether other age-related variables are more likely to explain the precision of NL estimation. To verify this, we performed a Bayesian analysis of covariance (ANCOVA) with CP-knowledge level as between-subject factor, age in days as a covariate, and PAE as the dependent variable. We decided to run the Bayesian analysis because we expected a null effect of age, assuming that the knowledge of counting principles is responsible for developmental effects. Partly contradicting these expectations, the largest Bayes factor was found for age (BF_10_ = 15.067), however, for the model containing both age and CP-knowledge level, BF_10_ was also large (BF_10_ = 8.866). The ratio of these two values is 1.7, which indicates a greater probability of the model explaining developmental changes as related to age only, but it does not allow to exclude the hypothesis that both age and CP-knowledge play some independent roles in the development of the NLE ability. While not in line with our expectations, these results are not surprising. First of all, the age of 3–5 years is the age of the most intense development of executive functions, which may significantly affect the stability and accuracy of the estimation (Kolkman et al., 2013b). Next, in the age range studied here, usually only some of the oldest children can count in the 1–20 range, which may lead to more precise scaling of the line even within CP-knowers group. In this case, however, CP-knowledge level should be the main factor in the estimation of lines limited by the numbers 1 and 9, because almost all CP-knowers are able to efficiently use numbers in this range. Analyses of segmented line suggested that this may be the case. Testing this directly was one of the goals of Experiment 2.

## Experiment 2: 1–9 number line oriented from left-to-right

The previous experiment showed that even the youngest and least numerically-competent preschool children have some preference for placing sets of different numerosities along number-lines according to the linear rather than the logarithmic or cyclic power models. However, the children’s estimates were imprecise (mean absolute error above 25% of the scale) and the model fits were moderate or low at the individual level. Children had also more problems with estimating the position of the numerosities near either end of the scale. All these issues may be somehow related to the construction of the task and materials. Firstly, the “plates” (circles containing sets) were large: their diameter was almost 3 times larger than the distance between two adjacent numbers in the linear model. The materials were presented on a relatively small (13-inch) computer display. This may be one of the causes of the lack of precision in estimating the appropriate set position on the line, especially near its ends, where the plates marking 1 and 20 were permanently displayed. Moreover, for each child, each numerosity, and each line orientation, there were at most two measurements, which did not allow the computation of intra-subject estimation variance.

Secondly, the largest numerosity on the scale was much greater than the exact numerical knowledge of the less numerically competent participants. This may partly explain why the exact age of the respondents (which is typically correlated with knowledge of the count-list) was a better predictor of results than CP-knowledge level. Perhaps for the same reason, the results of Experiment 1 were not fully conclusive as to whether the youngest participants used the homogeneous or multi-segment linear+log number-line model. Taking all this into account, we decided to design a modified task with a shorter numerical range (1–9) and to use a larger display (19 inches), which provided a much larger spatial distance between adjacent numerosities—both in absolute terms and relative to the diameter of the “plate.” Shortening the scale led also to a reduction of the number of numerosities to be tested, which in turn allowed the testing of each numerosity four times, still keeping the total number of trials not too tiring for children, which additionally should increase the reliability of the mean and variance of estimates of each numerical quantity. Because we found in the previous experiment that line orientation does not affect either estimation precision or model preference, only the canonical left-to-right oriented line was used this time.

### Methods

#### Participants

Sixty-four children recruited from two preschools in Warsaw, participated (34 female; age range: 4.01–5.99, *M* = 4.84, *SD* = 0.76). The same rules for obtaining consent to participate in the study and rewarding the participants were used as in Experiment 1.

#### Procedure

The materials, apparatus, and procedure of Experiment 2 were modeled on Experiment 1, with differences listed below.

An all-in-one computer with a 19-inch touch screen (1,366 × 760 pixel resolution) was used. The first two training series were the same as in Experiment 1, while the third series consisted of only one trial in which the line ends were marked with sets of 1 and 9 elements (the same as in the test series) and 5 constituted the test numerosity. Then the child performed 24 test trials in which numerosities of 2, 3, 4, 6, 7, and 8 were tested, each four times with four different color and spatial arrangements. The number 5 was omitted from the test, as it had been used previously in training.

Since the children participating in Experiment 2 were part of the sample of a wider study of numerical abilities, consisting of several sessions, the Give-a-number task was administered in an earlier session than the NLE task.

### Results

#### Give-a-number task

Thirty children (GaN range: 2–4, *M* = 2.93, *SD* = 0.79, age range: 3.01–5.95, *M* = 4.42, *SD* = 0.74; 19 female) were classified as subset-knowers and 34 children (GaN range: 5–10, *M* = 8.58, *SD* = 2.03, age range 3.97–5.99, *M* = 5.24, *SD* = 0.58; 16 female) were classified as CP-knowers.

### Line-estimation task

#### Linear and logarithmic model fit

As in Experiment 1, for each child we computed the best-fit regression models with mean estimate as a dependent measure for linear and logarithmic distributions of estimated numerosities. Coefficients of determination (*R*^2^) with added slope sign (plus/minus) were submitted to repeated-measures ANOVA with model type (linear vs. logarithmic) as a within-subject factor and CP-knowledge level (subset-knowers, CP-knowers) as a between-subject factor. One subset-knower with significantly negative slope was excluded. Only the CP-level factor was found to be significant [M*_R_*2_subset-knowers = 0.375, *SE* = 0.066, M*_R_*2_CP-knowers = 0.579, *SE* = 0.061, *F*(1,61) = 9.31, *p* < 0.004, η^2^_p_ = 0.132]. Neither the model effect nor the interaction of both factors were significant (both *F*s < 1); M*_R_*2_lin = 0.451, M*_R_*2_log = 0.444 in subset-knowers; M*_R_*2_lin = 0.708, M*_R_*2_log = 0.702 in CP-knowers).

**Figure 3:**
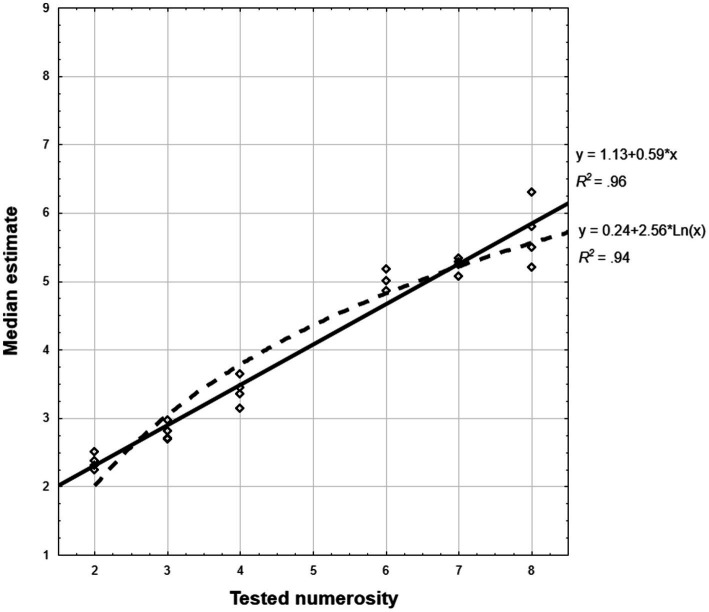
Experiment 2: 1–9 number-line: Distribution of linear and logarithmic model fit of group median estimates computed for each series separately. Regression equations and *R*^2^ coefficients for the best fit linear and logarithmic model are provided on the right margin.

Analyses based on group median estimates (computed analogically to Experiment 1) revealed high model fit. The linear model prevailed in entire sample [*R*^2^_lin_ = 0.962; *R*^2^_log_ = 0.938, *t* = −1.82, *p < 0*.05; see Figure 3], and CP-knowers [*R*^2^_lin_ = 963; *R*^2^_log_ = 0.941, *t*(23) = −2.07, *p < 0*.025], although did not reach statistical significance in subset-knowers [*R*^2^_lin_ = 0.942; *R*^2^_log_ = 0.904, *t*(23) = −1.29, *p = 0*.11; all tests one-tailed]. These effects were confirmed and also extended onto subset-knowers in supplementary analyzes in which children who showed negative regression slopes were excluded (Supplementary material). As in the previous experiment, the fit of the single-cycle power model with a free parameter across the entire sample fitted between the linear and logarithmic model (*R*^2^ = 0.942).

#### Scalar variance

The correlations of estimation variances with tested numerosity were significant and high, both for entire sample (*r* = 0.99, *p* < 0.001) and both CP-knowledge groups (*r*_CP-knowers = 0.97, *p < 0*.002, *r*_subset-knowers = 0.88, *p* < 0.025). In Supplementary material we show also analyses on the individual level. Four measurements collected for each numerosity allowed also individual variances to be computed. In such analysis the mean of these correlations was, unsurprisingly, much smaller than one based on entire sample, however, still significantly above zero [*M* = 364, *SE* = 0.053, *t*(63) = 6.83, *p* < 0.001, two-tailed one sample test), also after division into CP-knowledge-level groups CP-knowers: *M* = 0.491, *SE* = 0.060, *t*(33) = 8.20, *p* < 0.001, subset-knowers: *M* = 0.220, *SE* = 0.085, *t*(29) = 2.59, *p* < 0.015].

#### PAE analyses

Percent absolute error was significantly lower in CP-knowers (*M* = 19.17, *SD* = 7.79) than subset-knowers [*M* = 24.64, *SD* = 8.75, *t*(62) = 2.65, *p* < 0.011]. The mean PAEs here were lower than in Experiment 1, and similar to the results of earlier studies involving preschool children, even although those studies were usually conducted using symbolic or dual representations of numbers and with slightly older children.

#### Developmental effects

As the above analyses show, the precision of NL estimation was clearly higher than in Experiment 1 and it significantly depended on the level of CP knowledge. The results of Experiment 1 did not allow any conclusion to be drawn as to whether the CP-knowledge effect is actually caused by differences in numerical representations between CP-knowers and subset-knowers, or rather by more general age-related abilities. Analogously to Experiment 1, to test if the CP-knowledge effect observed both for PAE is specific to numerical knowledge development or more general developmental factors, we conducted a Bayesian ANCOVA with CP-knowledge level as the intergroup factor, age in days as covariant, and PAE as dependent measures. This time, the analysis showed CP-knowledge level effect as the most probable (BF_10_ = 4.598 for PAE; BF_10_ = 0.492 for age and BF_10_ = 1.332 for the model containing both Age and CP-level).

Altogether, the results of Experiment 2 confirmed and extended the conclusions from Experiment 1, supporting the linear model with scalar variance, rather than the logarithmic model, and evidencing the role of numerical knowledge development as a factor increasing the precision of non-symbolic NLE. These findings were additionally tested in Experiment 3 in which PN procedure was used.

## Experiment 3: Position-to-number task

Although the results of Experiments 1 and 2 together contradicted the log-to-linear shift hypothesis and provided some support for the linear model with scalar variance, it remains unclear whether mapping numerosities to positions on a line is driven by the inherent spatial properties of number representation, or rather by more flexible strategies. To provide a stronger test we designed the PN task with a 1–20 scale (the 1–9 scale used in Experiment 2 is too short to be used with the PN procedure). We replaced the traditional “production” version of this task (in which subjects estimate the number which matches a given position on a line), with its forced-choice version. The forced-choice PN task requires matching one of few deliberately selected numerical quantities to positions on the line, therefore, it is more determined. Moreover, the production task – traditionally used with symbolic numbers–in the case of non-symbolic materials it leads to different estimation biases (under-estimation and over-estimation patterns) than numerosity perception (Crollen et al., 2011).

The participant’s task was to choose from three options the one which corresponds to the position indicated on the line. One option was a “correct” (linear) numerical quantity corresponding to the marked position on the line, the other two were 0.67 and 1.33 of this value. We decided to use a constant ratio rather than values generated from logarithmic or exponential models, because in these models the distribution is too concentrated at one end of the scale (the small end for exponential models and the large end for logarithmic models). However, estimation based on a logarithmic model should increase the choice rate for too-small (0.67 × linear) numerosities and decrease the rate for too-large (1.33 × linear) numerosities (see figure in Supplementary material section D), and, in effect, the mean selected numerosity. This is because the PN task reverses the direction of the mapping, so the logarithmically scaled mental representation results in fitting to the exponential function. On the other hand, expected linear-model-based selection may range from equal distribution of all choices (assuming a high level of noise in estimation) to an elevated proportion of linear choices. In any case, the mean choices should, however, fit a linear distribution.

### Methods

#### Participants

Ninety-five children from three preschools participated (mean age = 4.29, *SD* = 0.57, range 3.21–5.48). Three additional children were removed from the sample because they did not follow the instructions. The same rules for obtaining consent to participate in the study and rewarding the participants as in Experiments 1 and 2 were used.

#### Materials, apparatus, and procedure

**Figure 4:**
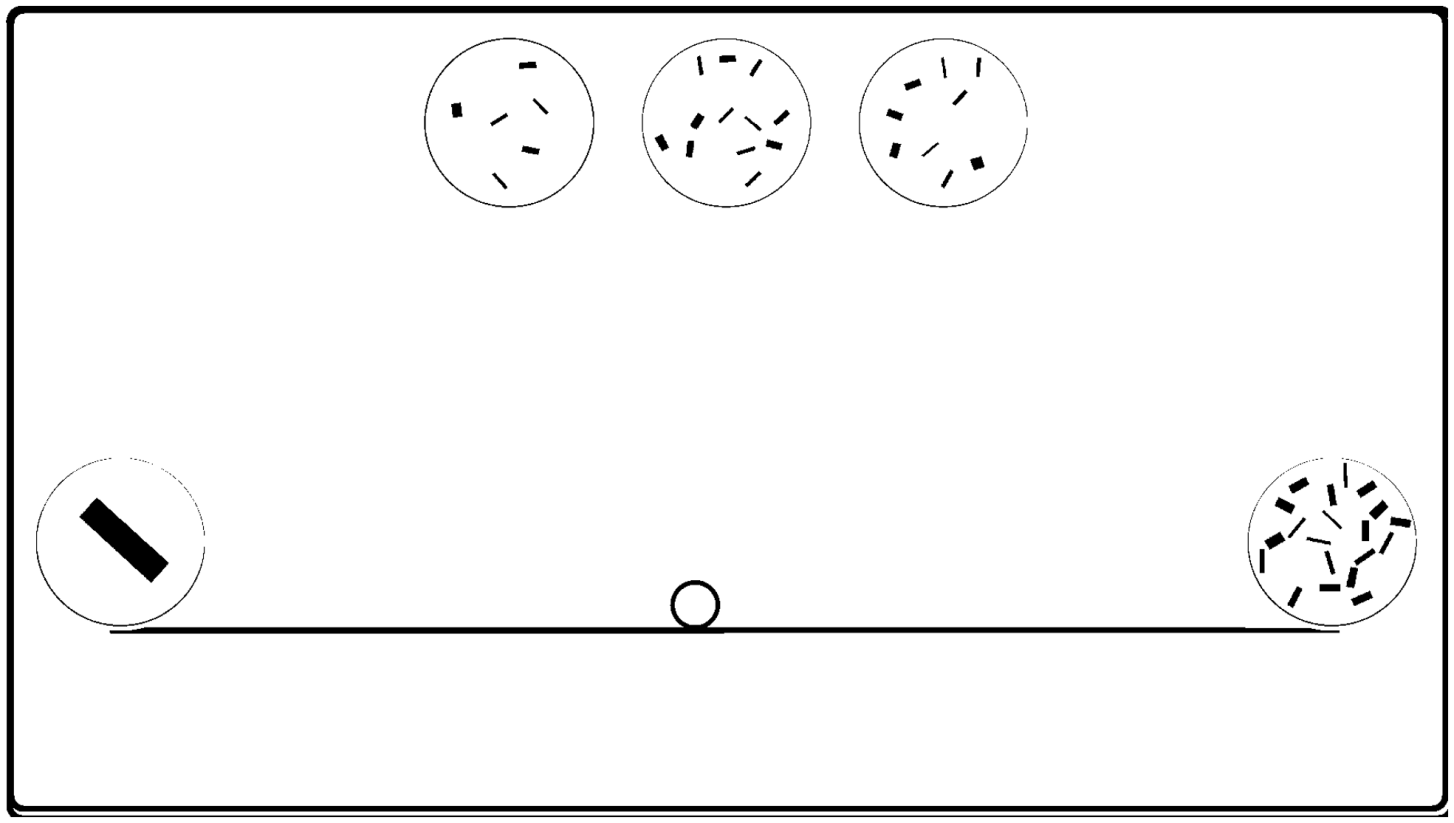
Experiment 3: Position-to-number procedure: Sample layout of the screen for the 6–9 – 12 test triplet. A small circle (“plate”) on the line indicates the position for which the appropriate set should be selected out of three options at the top of the screen.

The materials and apparatus from Experiment 1 were used in this study. The child first performed the give-a-number task and was then introduced to the number-line task. In the training session, a line limited by sets of 1 and 40 items in a left-to-right orientation was displayed on the screen, but this time there was a small circle (diameter of 40 pixels) in the middle of the line. The same story about the girl/boy placing plates of cookies was used and the child was informed that there was a small, empty plate without cookies and the protagonist should put some cookies on this other plate. At this point, a row of three sets (10, 20, and 30 items) in random order was displayed at the top. The child was asked which of these “plates” had the most and which the fewest “cookies” and was corrected if necessary. Then the experimenter asked which plate the protagonist should put in place of the empty plate and asked the child to indicate it by touching the screen. Regardless of the choice made by the child, the program placed a 20-element set in the middle of the line, and the experimenter said: “Yes, look, now this plate is standing here!” or “I guess not. Look! Ann/Tom put a different plate here.” The experiment then proceeded to the next trials, in which positions at 25 and 75% of the length of the line were tested. However, in the third training trial, the child was encouraged to complete the task on their own and the answer was not commented upon. After the training, the experimenter repeated the instructions and, having made sure that the child was ready, started the test trials. The test consisted of two series of 14 trials, using the line ending with numerosities 1 and 20 and the set of triplets listed in Table 2. The order of the trials within each series as well as the order of sets within a given triplet were random. Due to a programming error, the 11–15–20 triplet was correctly displayed only in the second series, so it was counted only once. Figure 4 illustrates test-screen arrangement.

**Table 2:** Triplets of numerosities tested in Experiment 3.

Choice options		
Below-linear ~N^*^0.*67*	Linear *N*	Above-linear ~N^*^1.*33*
1	2	3
2	3	4
3	4	5
3	5	7
4	6	8
5	7	10
6	8	11
6	9	12
7	10	14
8	11	15
9	12	16
10	13	17
10	14	19
11	15	20

### Results

#### Give-a-number task

Thirty-four children were classified as subset-knowers (mean GaN score: 2.35, *SD* = 0.74, range 1–4; mean age: 3.88, *SD* = 0.88, range: 3.23–5.20) and 61 were classified as CP-knowers (mean GaN score: 7.26, *SD* = 2.13, range: 5–10; mean age: 4.51, *SD* = 0.49, range: 3.21–5.48).

#### Position-to-number estimation task

##### Distribution of choices

In the first step, we checked whether the proportions of choices of the correct (linear) option in particular trials in the entire sample were higher than that expected from the random distribution (0.333). Contrary to expectations, the two-tailed single sample *t*-test showed that the linear option was chosen less frequently than it would be expected from the random distribution [M = 0.264, SD = 0.052, t(26) = −6.997, *p* < 0.001]. This result was then confirmed in one-tailed *t*-tests separately for subset-knowers [M = 0.308, SD = 0.075, t(26) = −1.740, *p* < 0.05] and CP-knowers [M = 0.237, SD = 0.066, t(26) = −7.390, *p* < 0.001]. In Table 3 we present the average proportions of the choices of each option for the entire sample and for subset-knowers and CP-knowers separately. We also provide the results of paired *t*-tests comparing the frequencies of linear option choices with too low and too high options.

**Table 3:** Mean percentage, standard deviations, and *t* values of choices of below-linear, linear, and above-linear options.

	Choice options			Paired *t*-tests between options	
**CP-knowledge level**	**Below-linear *M* (*SD*)**	**Linear *M* (*SD*)**	**Above-linear *M* (*SD*)**	**Below-linear vs. linear**	**Below-linear vs. above-linear**
Subset-knowers	32.8 (12.9)	30.3 (8.3)	35.3 (12.9)	*p* > 0.2	*p* > 0.4
CP-knowers	40.0 (21.8)	23.6 (12.1)	35.2 (20.9)	*t*(60) = −4.52, *p* < 0.001	*t*(60) = 0.91, *p* = 0.37
All	37.4 (19.3)	26.0 (11.3)	35.5 (18.4)	*t*(97) = 4.34, *p* < 0.001	*t*(94) = −0.59, *p* = 0.56

**Figure 5:**
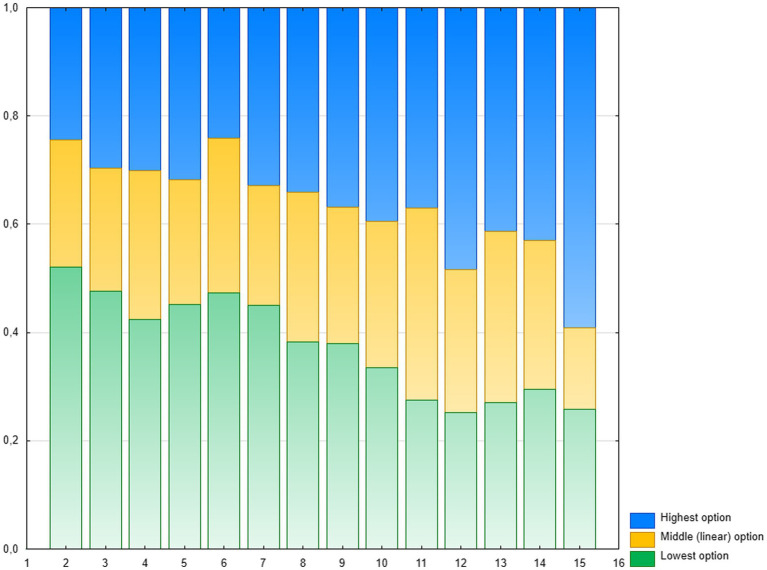
Experiment 3: Proportions of the choices of three options: the lowest (green), the middle–linear (yellow) and the highest (blue) for each position on the line.

One possible explanation of such a small proportion of the correct choices may be that children were selecting the options numerically most similar to that on the one end of the scale. This strategy was previously observed in Experiments 1 and 2, albeit not very often, as well as in other studies (e.g., Núñez et al., 2012; Sella et al., 2018). However, this strategy can be either categorical or scaled (based on numerical distance being a property of numerical representation). In the first case, for positions close to the ends of the line, the choices of the lowest or the highest numerosity (appropriately) should strongly prevail, with a certain range of uncertainty in the middle of the line, which would lead to a sigmoidal distribution. In the second case, assuming linear numerical representation, the proportion of choices below the linear option should decrease steadily with the position on the number-line, the proportion of choices above the linear option should increase, and the proportion of choices of the linear option should be more or less constant. The distribution of choices, visualized in Figure 5, shows a pattern congruent with this later expectation. The correlation of the mean proportion of below-linear choices with position on the line was strongly negative (*r* = −0.90, *p* < 0.001), with a fairly steep slope. For above-linear choices, the correlation was strongly positive (*r* = 0.82, *p* < 0.001) and steep. For linear choices, the correlation was weak and non-significant (*r* = 0.23, *p* = 0.22), with relatively flat slope. The same pattern of matching occurred both in the case of CP-knowers (*r* = −0.89 and *r* = 0.83, both ps < 0.001 for below-linear options and above-linear options, *r* = 0.20, n.s. for linear option) and subset-knowers (*r* = −0.54, *p* < 0.002, *r* = 0.35, p < 0.05, and *r* = 0.11, n.s. respectively, one-tailed tests), however, the dispersion of choices, was much greater in subset-knowers, and thus the precision of matching was significantly greater in CP-knowers, both in the case of above-linear (*p* < 0.01) and below-linear (*p* < 0.02) options. Thus it seems that probability of the choices based on similarity to line ends is linearly dependent on numerical distance between the option and the anchor (end point), and, as in previous experiments, the precision of fitting the linear model increases with CP-knowledge level.

In Supplementary material (part D), we present graphs illustrating the fit of mean selections to the linear and logarithmic model depending on the CP-knowledge level. In all cases, the R^2^ value of the linear model is higher than of the logarithmic one. However, it should be remembered that the applied procedure forces an increase of the mean of the selected option along with the numerical value corresponding to the indicated position on the line, therefore such an analysis has limited credibility. We present it only for illustrative purposes.

#### Developmental effects

Bayesian ANCOVA was run on the individual linear model *R*^2^, based on mean selected numerosity from two runs with CP-knowledge level as the between-subject factor and age as covariate. The largest Bayes factor was found for CP knowledge (BF_10_ = 22.012). Also, for the CP knowledge x age interaction, the indicator exceeded the value of 3 (BF_10_ = 7.615), while for age itself, it was below 3, thus failing to confirm that age, independent played any important role in the precision of estimation. Meanwhile, the results of Experiment 3 again support the hypothesis of the linear model already in the youngest and least numerically competent participants, with an increase in the precision of estimation associated with the acquisition of numerical competences.

## General discussion and conclusion

The purpose of this series of experiments was to investigate the extent to which the non-symbolic NLE task can reveal the most basic properties of number representation, including its spatial properties, the development of this representation, and its dependence on the child’s mastery of symbolic numerical representations. Our goal was also to fill a gap in the research, as there has thus far been very little use of non-symbolic NLE tasks, and almost no studies investigating children aged 3–5 years old (none using a PN procedure).

In Experiment 1, we found no relationship between performance on the NLE task and left-to-right or right-to-left line orientations. The linear model was a slightly, but significantly, better fit than the logarithmic one; however, the children’s distribution of the sets on a 1–20 line turned out to be generally inaccurate and relatively poorly matched to both models at an individual level. Both accuracy of estimation (PAE) and fit to either mapping functions increased significantly with the level of mastery of counting principles, although this effect may be due to other age-related differences. Importantly, although we tested different models, including segmented number-line or one- and two-cycle power models, participants appear to have treated the entire numerical range as a whole, rather than using separate mapping functions or strategies for individual ranges, such as within subitizing range or for the most extreme large numbers. Finally, the correlation of the estimation variance with the estimated numerical values was weak not providing considerable support for the model of linear numerical representation with scalar variance.

In Experiment 2, in which a 1–9 line oriented from left to right was used, we obtained much greater mapping accuracy and better fit for both main modeling functions (logarithmic and linear). The fit increased specifically with the level of mastery of counting principles (but also with age). The significance of the prevalence of the linear model over log and cyclic power ones got clear support only in the group median analysis. This time, however, the variance of estimation significantly increased with the numerical quantities, as predicted by linear model with scalar variance. There was a developmental progression in PAE, this time being related to the knowledge of counting principles rather than other age-related factors.

In Experiment 3, we used a PN version of the NLE task. The main result was a strong effect of anchoring or similarity to numerosities at the ends of the scale. Up to halfway on the scale, the smallest set was chosen most often, while from halfway onward the largest set was chosen most often. Strength of these tendencies was, however, linearly correlated with numerical distance between the anchor and selected option, which supports linear model. That was further supported in the analysis of the individual fits of mean choices to log and linear models.

Below, we discuss these results in the context of the three main theoretical issues underlying our research: (1) the role of line orientation, (2) the type of model which best explains the estimation of the position of a number on the axis, and whether performance on the NL task directly reveals the scaling and spatial properties of the mental representation of number, and (3) developmental change in non-symbolic NLE.

### Line directionality

In some contrast to the results of the only studies known to us in which the orientation of the number-line was subject to experimental manipulation or measurement (Ebersbach, 2015; Sella et al., 2019, 2020), the results of Experiment 1 quite unambiguously identified a lack of dependence on the line directionality of both estimation accuracy and mapping function. Due to the robustness of this result, we abandoned the manipulation of line direction in subsequent experiments. What makes our study different from those of Sella et al. (2019, 2020) is the use of non-symbolic materials and the direct manipulation of line orientation (in the studies of Sella et al., the children freely selected the direction of the mapping). On the other hand, Ebersbach (2015) used two formats to present numbers (dot collections and spoken number words) as well as a much larger numerical range (1–100); additionally her participants were older (6–8 years of age). Thus, our study was the only one in which only non-symbolic representations of a number were used in the early development of numerical concepts. We can argue carefully that the orientation of the line is not a factor which affects the most basic operations of estimating the position of a numerosity on a line, although it may play a role in the case of the symbolic representations of numbers and their ordinal properties.

At the same time, a number of other studies have shown a close relationship between the representation of numbers and spatial directions, also in preschoolers and in even younger participants (Patro and Haman, 2012; McCrink and de Hevia, 2018, for review). Moreover, some of these studies used a numerosity comparison task involving similar materials and were carried out on a sample from a similar population as in this research. This may mean that the distribution of numbers along the line may rely on processes other than using the spatial properties of the mental representation of the numbers to compare them. As pointed out by Patro et al. (2014), space is a multifaceted concept which may refer to either spatial directions or to spatial extensions (area or length), which are not directional *per se*. Both forms of mapping may exist in young children, but they may be activated by different tasks.

Alternatively, it is also possible that young preliterate children are able to flexibly switch between left-to-right processing (as default) and (optionally) right-to-left number processing to optimize their performance on a given task. If this explanation is true, it would mean that the directionality of a line which serves as a basis for numerical estimation may become more crucial for participants mastering symbolic representations (Sella et al., 2019, 2020) or who are more used to number ordering conventions (Ebersbach, 2015), although plasticity of task-dependent directional spatial-numerical associetions was reported also in adults (Fischer, 2006).

### Comparison of the linear and logarithmic models

The second issue which we wish to discuss concerns the models of basic numerical representation (mental number line). Let us assume for a moment that the NL estimation task is a reliable test of the internal number representation model (we will loosen this assumption later in the discussion). The analyses carried out in all three experiments indicated that the linear model better explained the empirical data, although not all measures showed a significant advantage of the linear model in every sub-sample (subset knowers subgroup in Experiment 1, individual fit analyzes in Experiment 2). Additionally, Experiment 2 (but not Experiment 1) provided strong evidence for the scalar variance of estimates, which is expected in an uncompressed linear model. It is worth noting that although the logarithmic mapping of the sets’ numerosities to a line was found in the study by Dehaene et al. (2008), and as an initial model in studies by Siegler and Opfer (2003) or Kim and Opfer (2018), the few other studies that used non-symbolic materials, especially those involving young children, showed mixed results, with the advantage of the linear model (either unitary or multi-segment; e.g., Ebersbach et al., 2008; Sasanguie et al., 2012b), at least in some numerical ranges, which is in line with our results. The question arises as to why non-symbolic NLE in children does not decisively fit the log-to-linear shift found in the symbolic number-line task. In the first step, we must eliminate the possibility that children’s estimates were guided by continuous spatial (e.g., cumulative area, density, convex hull etc.) rather than numerical cues (*cf*. Sella et al., 2015). We tried to prevent this by generating sets with various combinations of spatial parameters. Unfortunately, this solution works effectively with a large number of trials, while studies with young children require minimizing the testing time and reducing the number of trials. It is also unclear whether visual numerosity coding is possible at all without parallel coding of correlated spatial cues (Lourenco and Aulet, in press) However, as shown by Cicchini et al. (2016), spatial cues play a greater role in the cases of large numerosities and high density of set elements, which is not our case. Also, scalar variance of estimates seems to be at odds with this explanation.

A possible alternative is that elementary mental representations of numbers are not logarithmically scaled. For example, the accumulator model of Meck and Church (1983) considered in Siegler and Opfer (2003) and Ebersbach et al. (2008) assumes scalar variance without logarithmic compression. The results of Experiment 2 support this explanation, given that the estimation variance was strongly correlated with the cardinality of the estimated set. Current knowledge of neural numerical codes in the parietal cortex also seems to be more compatible with such a model (Piazza et al., 2004; Nieder and Dehaene, 2009). As some computational models suggest, logarithmic scaling of number representation may not be so much an inherent property of number representation, but rather the result of “on-line” number-to-space mapping processes using “recycled” spatial attention mechanisms adapted for arithmetic and other numerical operations (Chen and Verguts, 2010). Logarithmic scaling and log-to-linear shift found also in some studies using non-symbolic task may be related to the stimuli used there: large numerosities (up to 100 or more) which result with high spatial density. Another explanation points to using zero as the lower line delimiter, which may not be scaled in the same way as positive numbers – this may lead to illusory logarithmic scaling.

Still another alternative is that logarithmic compression may be an inherent property of primitive representation of numbers, however, the processes of mapping numbers onto a line do not directly rely on these representations, but are based on other relations. The models which contradicted the hypothesis of log-to-linear shift, such as proportional reasoning (Barth and Paladino, 2011), or ordinality based models (Sella et al., 2020) or other indirect estimation strategies (Karolis et al., 2011; Cohen and Sarnecka, 2014) are not necessarily at odds with this explanation. It should be noted here that recoding a logarithmic scale with a constant variance to a linear scale will result with a scalar variance of the final code. The claim about contextual and strategic variability of mapping numbers onto line is well established empirically in research on symbolic NL. Already in the study by Opfer and Siegler (2007), the fit of the mapping changed after one-time feedback correcting the linear position of the number. Dynamic nature of mapping strategy shift was also shown by Kim and Opfer (2018) in children with non-symbolic task. In our Experiments 1 and 2, the children received training in which the position of the set was corrected according to the linear model, although the justification for shifting the set referred only to ordinal (not scaling) relations. However, the problem with the explanation assuming logarithmic compression of the basic numerical representation and flexible mapping strategies is that in such a case NL estimation task cannot be treated as a method of reliably discovering the intrinsic properties of the basic representation.

Thus, we come to the question of to what extent the NLE task is able to directly reveal the properties of the mental representation of numbers. Originally it was assumed that since the representation of a number has spatial properties and takes the form of a mental number-line or a “mental ruler,” these properties should directly transfer to the visualization of numbers on a physical number-line. As first documented by Siegler and Opfer (2003) and Siegler and Booth (2004), the log-to-linear shift matched quite well what was known about the development of number concepts. Recent studies by Kim and Opfer (2018) and Lee et al. (2022) also provides support for this thesis. However, there are several works suggesting a multitude of parallel mappings (separate linear segments with different slopes for numbers within and outside the range of mastered numeral meanings; see Ebersbach et al., 2008; or units and tens; see Moeller et al., 2009). Also, manipulating a reference number in memory can induce linear or compressive scaling, as shown by Lourenco and Longo (2009) in the line bisection task. This may indicate that the process of mapping numerical magnitudes onto external spatial representations can be more complex than directly copying the inherent spatial properties of internal representations. The same conclusion can also be drawn from studies which demonstrate the role of the anchoring effect and references to previous trials (Karolis et al., 2011; Cicchini et al., 2014; Sullivan and Barner, 2014). Also, the results of our Experiment 1 indicated that children have trouble mapping numerosities close to the ends of the line, while Experiment 3 indicated a strong anchoring effect associated with the line ends. All this suggests that the spatial scale for numbers in the NE task is constructed on-line and contextually, rather than reflecting a fixed internal “number space.” The work of Sella et al. (2018, 2020), show one more aspect of this problem. At least in the case of symbolic numbers, a full understanding of their ordinal properties (predecessor and successor functions) and of comparing numerical quantities comes with the ability to map numbers to a line later than formally reaching the CP-knowers level. Subset-knowers and CP-knowers who have not yet reached this stage in an NLE task usually locate numbers using an end-point or axis-center anchor strategy. It cannot be ruled out that full understanding of ordinality, originally acquired along with the concept of an exact number, is then also projected onto the approximate number system.

The question arises why our results differ so radically from the results shown in some other studies, and especially in the study by Kim and Opfer (2018), whose procedure and materials (Experiment 4, non-symbolic estimation in children) were largely similar to our Experiments 1 and 2. While there is no conclusive evidence here, it is possible that it is due to use of zero as the line’s lower bound. As mentioned previously, it is far unclear whether zero is directly represented in the ANS. In particular, it seems that until school age children are not able to decide which set, one-element or empty set, is smaller (Merritt and Brannon, 2013). If zero is not part of the basic system of representation of numbers in children then the zero-based scale is poorly defined for the child. Indeed, children may have a tendency to place any numerosities closer to the end labeled with non-empty set, thereby compressing their estimates. On the other hand, the use of zero may promote proportional reasoning especially in more numerically competent subjects, which would explain why proportional models fitted worse than linear models in our study, but was prevailing in some other studies.

In summary, our research add to those demanding a cautious approach to interpreting the results of the NLE task as directly indicating the structure of internal numerical representations. This conclusion goes in line with that concerning the lack of a number line directionality effect. Together, they suggest that mapping numerical quantities to the linear spatial representation may reflect not so much the constant spatial properties of the numerical representations as a task-related *ad hoc* construction.

### The nature of the developmental change in number-line estimation

While the results of the current study provide some evidence against the log-to-linear developmental shift in non-symbolic numerical representation, we identified clear developmental effects on the precision of estimation. Unfortunately, our procedure does not make it clear whether it is domain-specific or domain-general dependencies. In all three experiments, CP-knowers showed a higher precision of mapping. However, only in the Experiments 2 and 3, additional analyzes allowed to state that the level of CP-knowledge is of greater importance here than the general developmental factors related to age. But even in this case, we cannot rule out that some more specific, but still non-numeric individual differences, e.g., some kind of executive or spatial skills, affect both the acquisition of the counting principles and the mapping of numbers onto the line. Nevertheless, the possibility that the precision of NLE is partly dependent on symbolic numerical knowledge (CP knowledge) is still worth considering One may speculate that abilities or knowledge acquired with symbolic representation, such as the principles of successor or the constant unit, can also be used in the processing of non-symbolic numbers, leading to a more stable scaling of the line. The two-way, rather than one-way, relationship between the non-symbolic (basic) and symbolic (cultural) representations of numbers is being increasingly considered in contemporary works (starting with Gallistel and Gelman, 1992; but see also Goffin and Ansari, 2019). Studying such bi-directional interdependencies between the non-symbolic and symbolic numerical systems may be one of the most promising directions for further research into the early development of numerical cognition.

## Conclusion

The results of the current study seem contradict the “log-to-linear shift” hypothesis within the primary number representation, rather supporting a linear model with scalar variance. Interestingly, they also indicate no importance of line orientation (left-to-right or right-to-left). However, they show a strong trend toward an increase in the precision of estimation (expressed both in the absolute error of estimation and model fit coefficients) along mastering the concepts of number and counting (CP-knowledge). While it is still unclear whether the NLE task actually measures the properties of the basic numerical representations, it seems that our results fit well with the research trend, in which the spatial aspects of number representation are task-dependent, on-line constructions, rather than inherent properties.

## Data availability statement

The raw data supporting the conclusions of this article will be made available by the authors, without undue reservation.

## Ethics statement

The studies involving human participants were reviewed and approved by Komisja Etyki, Wydział Psychologii, Uniwersytet Warszawski. Written informed consent to participate in this study was provided by the participants' legal guardian/next of kin.

## Author contributions

MH designed the main assumptions of the study, prepared E-Prime scripts, performed statistical analyzes, prepared the first version of the manuscript, and edited its final version. KP participated in the design of the experiments, generated research materials, and participated in the discussion of the results, and is the author of parts of the revised manuscript. All authors contributed to the article and approved the submitted version.

## Funding

This study was supported by National Science Center (NCN), Poland by grant nos. 2012/05/B/HS6/03713 (Experiment 1) and 2014/13/B/HS6/04072 (Experiment 2 and 3) to MH.

## Conflict of interest

The authors declare that the research was conducted in the absence of any commercial or financial relationships that could be construed as a potential conflict of interest.

## Publisher’s note

All claims expressed in this article are solely those of the authors and do not necessarily represent those of their affiliated organizations, or those of the publisher, the editors and the reviewers. Any product that may be evaluated in this article, or claim that may be made by its manufacturer, is not guaranteed or endorsed by the publisher.
