# Supplementary material for: More linear than log? Non-symbolic number-line estimation in 3- to 5-year-old children
Source: Front Psychol. 2022 Oct 31;13:1003696. doi: 10.3389/fpsyg.2022.1003696 (PMC9659870; doi:10.3389/fpsyg.2022.1003696)

**A. Experiment 1. Comparison of linear and logarithmic model excluding children with a negative regression slope**

As noted and justified in the main text, when comparing the individual fit of the models, we used *R*^2^ with the added regression slope sign (plus or minus) rather than rejecting children with a negative slope as outliers (as done in previously published studies). However, this method of analysis, although justified in our study, impairs comparability of our results with previously published ones. Here we present analyzes involving only those children who showed a positive regression slope. We merged both CP-knowledge levels and ran a paired *t*-test in this case, rather than an ANOVA, because 16 children with negative slopes, mostly from the subset-knowers group, had to be removed from this analysis. An analysis divided into CP-knowledge levels, at least in the case of subset-knowers, would be unreliable due to too small sample. The advantage of the linear model over the logarithmic model was again confirmed (*M*_lin_ = .404, *SD* = .321, *M*_log_ = .358, *SD* = .293, *t*[46] = 4.26, *p* < 0.001).

**B. Experiment 1. Graphs presenting distribution and fit of group median estimates to logrithmic and linear models with division onto line orientation and CP-knowledge level**

In the figures below, the distribution of the median estimates and their fitting to the linear and logarithmic model is visualized with the division into CP-knowledge level (subset knowers and CP-knowers) and line orientation (left-to-right and right-to-left). Regression equations and *R^2^* coefficients of the best-fitted models are included. Note that in line with the general analysis presented in the main text, none of the differences for the subset-knowers reached significance (both *p* > .4). There are also no significant difference between the quality of fit of a linear or logarithmic model depending on the orientation of the lines.


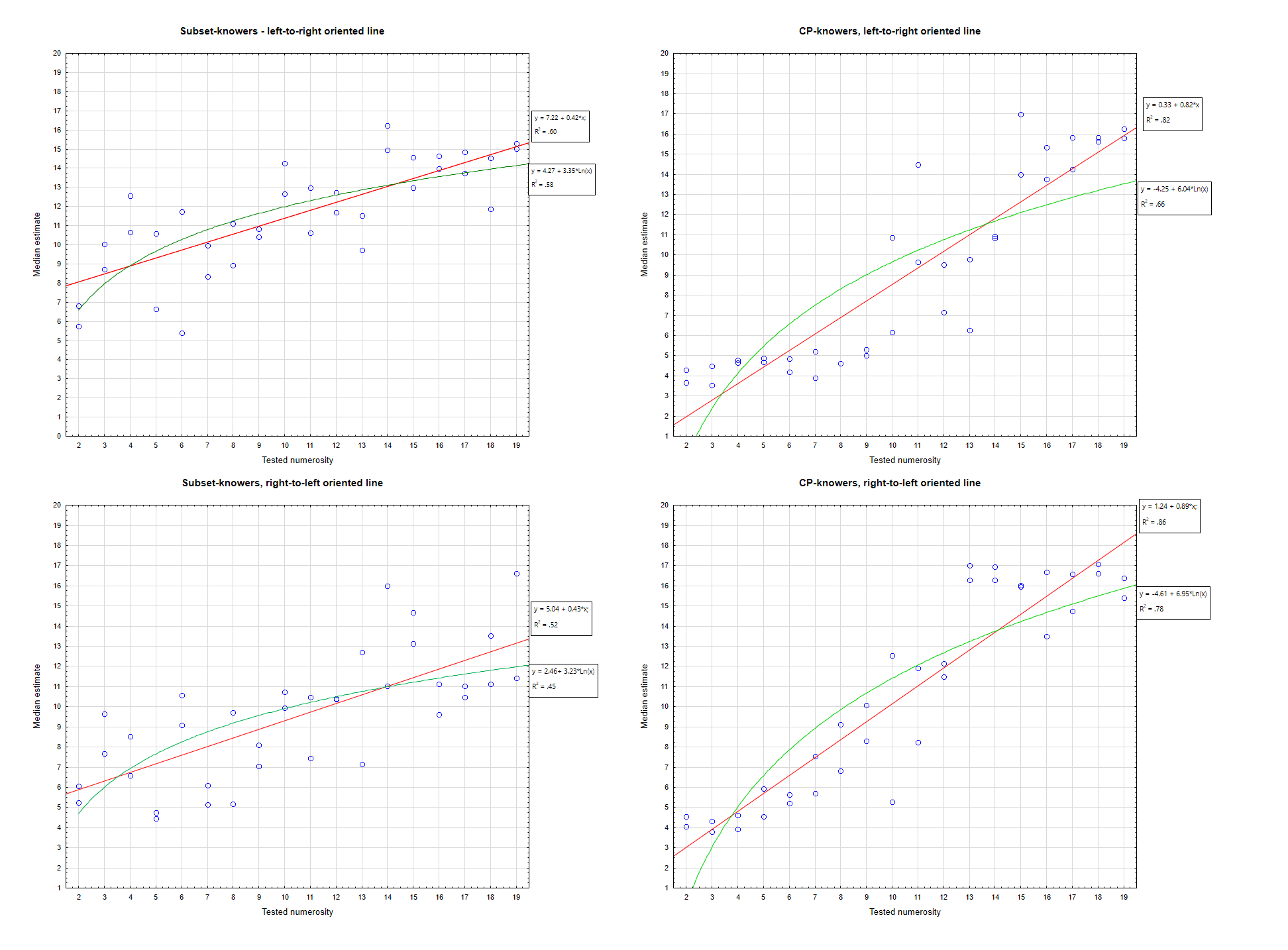
**C. Experiment 2. Comparison of linear and logarithmic model excluding children with negative regression slopes**

Six subset-knowers and one CP-knower displayed negative slopes of regression of estimated positions on the line in Experiment 2. We repeated the CP-knowledge level (2: subset-0knowers vs. CP-knowers) x Model (2: linear vs logarithmic) ANOVA on individual model fit (*R2*), removing the data collected from these children. The results almost perfectly replicated the previous analyses (*F*[1, 55] = 5.38, *p* < .025 for the CP-knowledge level factor and *F*s < 1 for the model factor and interaction), except that for both models the mean fit increased, especially in the subset-knowers group, which is purely a formal effect.

For group medians distributions, linear model was better fit than logarithmic model for entire group, as well as for both CP-knowledge level separately (Linear model: entire group *R2*=.95, Subset-knowers *R2*=.92, CP-knowers *R2*=.96; logarithmic model: entire group *R2*=.92 all, Subset-knowers *R2*=.86, CP-knowers *R2*=.94). Series of paired *t*-tests on absolute values of regression rests (cf. Siegler & Booth, 2004) revealed that all these differences, including subset-knowers group are significant, which supports and further strengthens the results of the analysis carried out without eliminating children with negative slope (entire group: *t(23)*=-3.95, *p*<.001; Subset-knowers: *t(23)*=-4.00, *p*<.001; CP-knowers: *t(23)*=-2.10, *p*<.025, all tests one-tailed).

It should be noted that in the full-group subset-knowers analysis reported in the main text, the difference in the fit of the linear and logarithmic models was not significant. A clear significant difference in subset-knowers after eliminating children with negative slopes may indicate that even the least numerically competent children estimate the position of a set of a given numerosity on the 1–9 number-line using a linear model. It is likely that six rejected children with negative regression slopes either pointed to the positions on the line at random or were children with a strong preference for right-to-left line orientation, resulting in a more negatively sloped regression for the linear model than for the log model.

**D. Experiment 3. Graph comparing different model fit of mean selected number-option in both CP-knowledge level groups**

The figure illustrate how average numerical values of selected options fit linear and log models (exponential function was used to model logarithmic choices). *R^2^* coefficients are provided as a measure of the model fit.


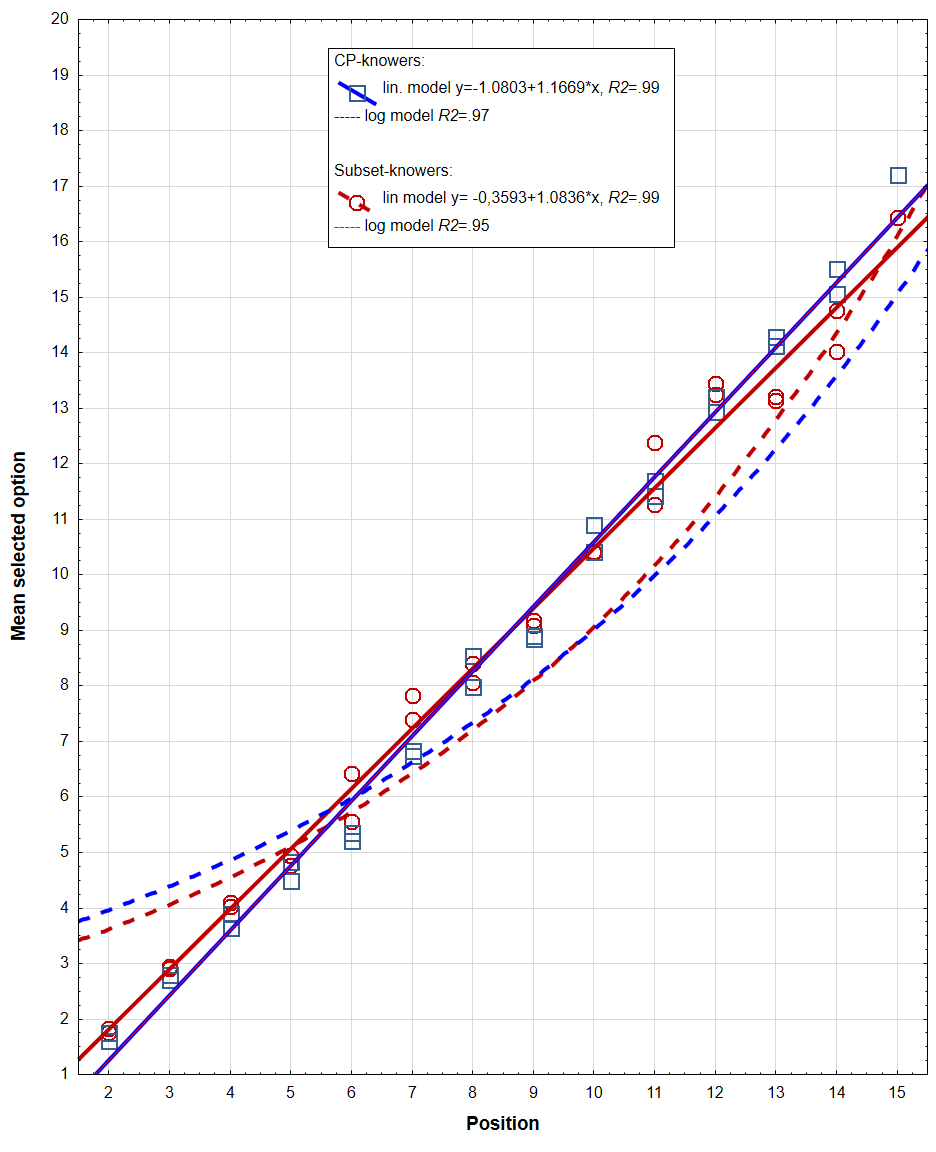

Supplement: Supplementary file 1 [file Data_Sheet_1.docx]
